# Desmoid tumor of the abdominal wall: a case report

**DOI:** 10.1186/1752-1947-5-326

**Published:** 2011-07-25

**Authors:** Athanasios Economou, Xanthi Pitta, Efstathios Andreadis, Leonidas Papapavlou, Thomas Chrissidis

**Affiliations:** 1 Department of General Surgery, General Hospital of Edessa, Terma Egnatias 58200 Edessa, Greece; 2 Department of Radiology, General Hospital "Agios Pavlos," Ethn. Antistaseos 161, 55134 Thessaloniki, Greece; 3 Laboratory of Pathology, General Hospital of Edessa, Terma Egnatias 58200 Edessa, Greece

## Abstract

**Introduction:**

Desmoid tumors are rare lesions without any metastatic potential but a strong tendency to invade locally and to recur. These tumors are associated with women of fertile age, especially during and after pregnancy.

**Case presentation:**

The case of a desmoid tumor of the anterior abdominal wall in a 40-year-old Caucasian man with no relevant family history is presented, describing its appearance on computed tomography and ultrasonography. The patient, who presented with a painless mass in the left anterolateral abdomen, had a history of previous urgent abdominal surgery after a shotgun injury two years earlier. Radical resection of the affected abdominal wall musculature was performed, and the defect was reconstructed with polypropylene mesh.

**Conclusion:**

The diagnosis of desmoid tumor should be strongly considered even in male patients with an abdominal mass and a history of previous abdominal surgery. The goal of its treatment is complete tumor excision and avoidance of the development of complications such as hernia.

## Introduction

Desmoid tumors are histologically benign neoplasms with a strong tendency to recur locally after resection and account for 0.03% of all neoplasms and 3% of all soft tissue tumors. These tumors have an intermediate biological behavior between benign fibrous lesions and fibrosarcomas. They occur usually between the ages of 25 and 40 years with a strong prevalence among women in the fertile age group. The most common site of predilection is the anterior abdominal wall, with an incidence of 50% [1-8].

We present the case of this rare medical entity in a 40-year-old man with history of abdominal surgery and describe its appearance on computed tomography (CT) and ultrasonography.

## Case presentation

A 40-year-old Caucasian man presented to the emergency department with a painless mass in the left anterolateral abdomen. During a physical examination, the mass was firm, nontender and fixed to the abdominal wall. The patient stated that the mass was gradually increasing in size. He had no relevant family history and did not smoke, drink alcohol or take any medications. Analyzed blood parameters were within the normal range, and tumor marker results were negative. The patient had a history of previous urgent abdominal surgery for traumatic rupture of the left colonic flexure and part of the small bowel after a shotgun injury two years earlier.

**Figure 1:**
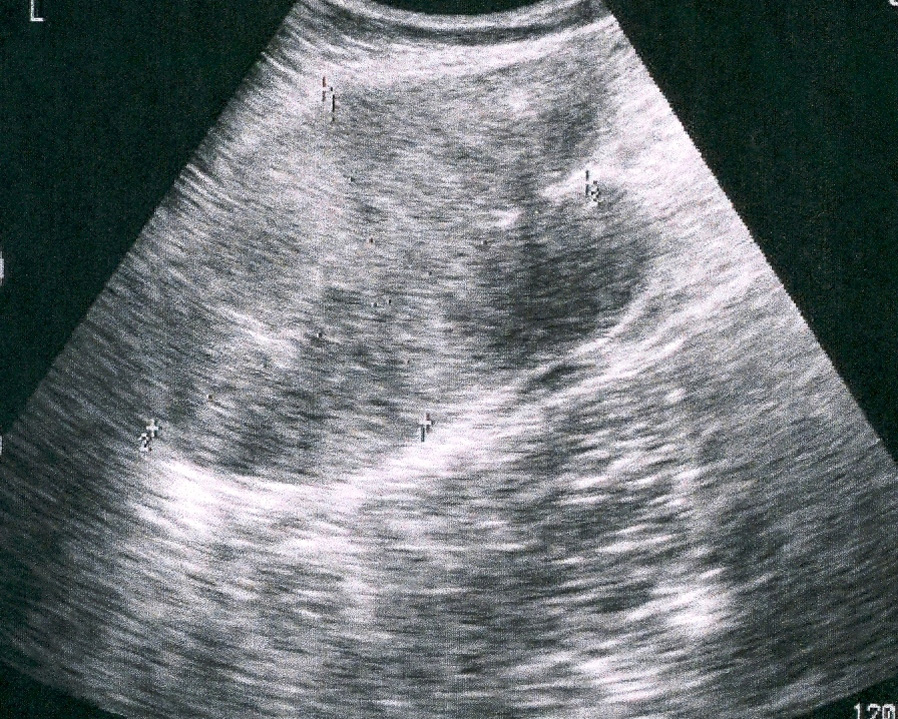
**Ultrasound image showing a large mass of heterogeneous echogenicity with smooth, sharply defined margins**.

**Figure 2:**
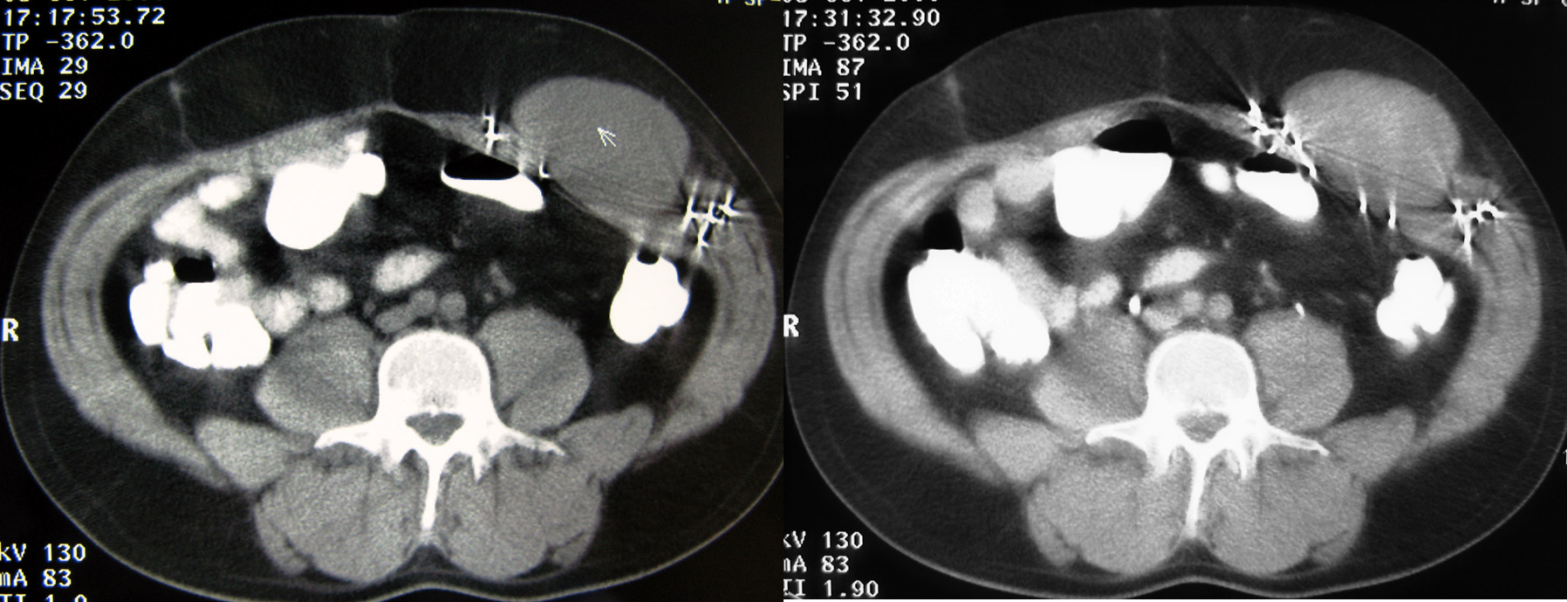
**Axial CT scan images of the abdomen**. Computed tomography examination before (**A**) the intravenous administration of contrast medium revealing a well-circumscribed mass originating from the left rectus abdominis muscle and of attenuation equal to that of the muscle. The mass demonstrates a mild enhancement even in the delayed images after the intravenous administration of contrast medium (**B**). The presence of multiple foreign bodies caused by the shotgun injury.

An ultrasound examination was performed and demonstrated a large mass of heterogeneous echogenicity with smooth, sharply defined margins in the left anterolateral abdominal wall (Figure 1). Preoperative CT scan images revealed a well-circumscribed, large mass (9 × 8 × 6 cm) of attenuation equal to that of muscle. The mass originated from the left rectus abdominis muscle and after intravenous administration of contrast medium demonstrated mild enhancement even in the delayed images. No pathologic adenopathy was present (Figure 2).

**Figure 3:**
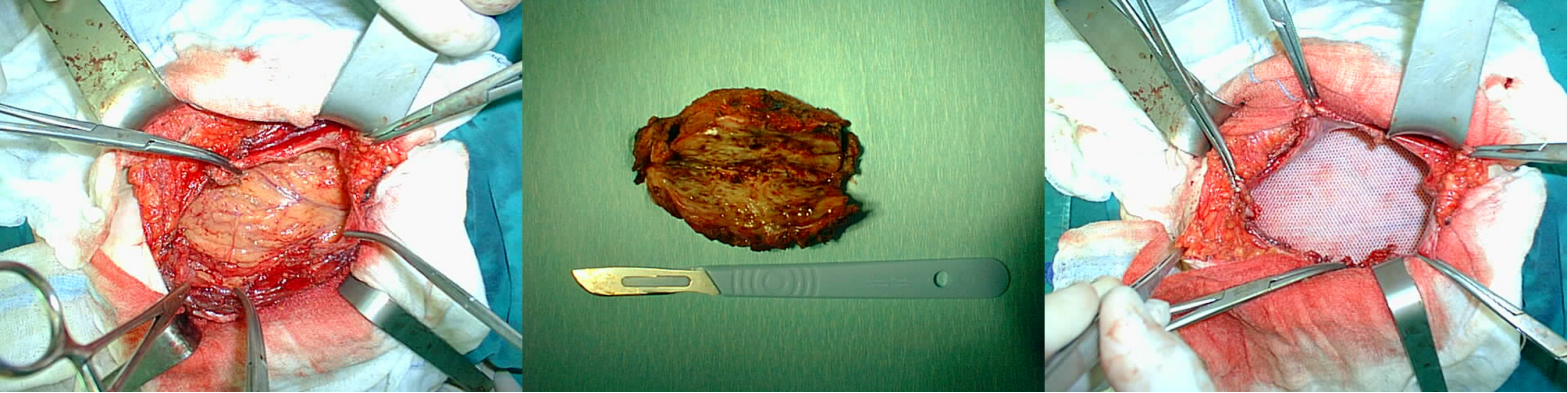
**Intraoperative pictures of surgery for abdominal wall desmoid tumor**. **A) **Abdominal wall with tumor. **B) **Macroscopic view of the tumor. **C) **Abdominal wall after polypropylene mesh repair.

**Figure 4:**
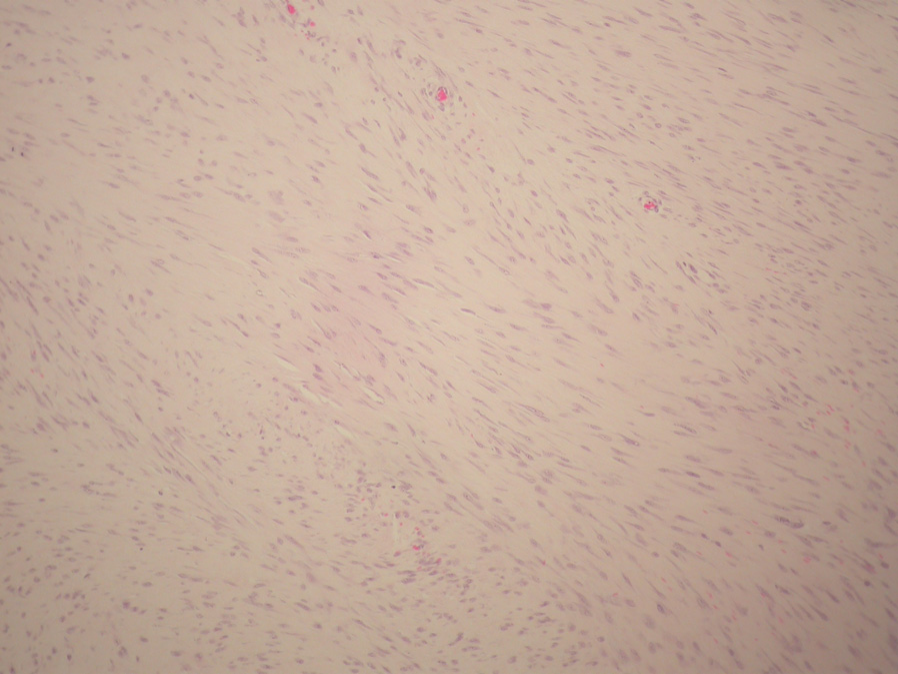
**Microscopic view of the excised rectus desmoid tumor showing fascicles of fibroblastic spindle cells with abundant intercellular collagen**. (Hematoxylin and eosin stain; original magnification × 200.)

Radical resection of the affected abdominal wall musculature down to the peritoneum was performed to include a peripheral margin of 3 cm of healthy tissue. The defect was reconstructed with polypropylene mesh. Macroscopically, the lesion had a firm, gritty texture. On the cut surface, it was glistening white and coarsely trabeculated, resembling scar tissue (Figure 3). The histologic diagnosis was of desmoid tumor (Figure 4).

The postoperative course was uneventful, and the patient was discharged on the sixth postoperative day. The patient remained well at three years of follow-up with no evidence of tumor recurrence or development of incisional hernia.

## Discussion

Desmoid tumor, also known as aggressive fibromatosis, is a rare tumor. Approximately 3.7 new cases occur per one million persons per year and develop mostly as an extracolonic manifestation of familial adenomatous polyposis (FAP) [1-8]. They differ from fibrosarcomas in the fact that despite their aggressive local infiltration, desmoid tumors do not metastasize to other parts of the body [4-6].

They can be divided into five subgroups: extraabdominal, intraabdominal, multiple, multiple familial and as part of Gardner's syndrome. Extraabdominal desmoid tumors have a wide distribution; the shoulder girdle, trunk and lower extremities are most commonly involved. Abdominal desmoids, which may occur in the abdominal wall, mesentery or retroperitoneum, have an increased incidence in individuals with Gardner syndrome. The histologic findings in these lesions are identical [1,5,6,8].

Abdominal wall desmoid tumors arise from musculoaponeurotic structures of the abdominal wall, especially the rectus and internal oblique muscles and their fascial coverings, and occasionally cross the midline. Less commonly, they originate from the external oblique muscle and the transversalis muscle or fascia [7].

The commonest groups associated with these tumors are young women during or after pregnancy. The fibroblast has been shown to exhibit a proliferative response to estrogen. Women with desmoid tumors have regression of their lesions after attaining menopause [1-9].

There is a well-known association in patients with a history of abdominal or pelvic surgery. This tumor is also associated with trauma, estrogen therapy, FAP and Gardner syndrome [1,4-6]. In fact, even though desmoid tumors are rare in male patients, in our case, the history of previous surgery, the location of the mass and the imaging features made its diagnosis possible.

Abdominal desmoid tumor usually presents as a mass that is sometimes associated with pain and weight loss [6]. Most of the abdominal wall desmoids measure 5 cm by 15 cm in diameter. Our patient presented with a painful mass measuring 9 cm in maximal diameter. These masses have a firm, gritty texture. On the cut surface, they are glistening white and coarsely trabeculated, resembling scar tissue. These tumors have no distinct capsule, and their margins are ill defined even when they appear well circumscribed on imaging [7].

The differential diagnoses for rectus abdominis lesions include acute hematoma, fibrosarcoma, lymphoma, rhabdomyosarcoma, liposarcoma, leiomyosarcoma, neurofibroma, benign fibrous tumor and primitive neuroectodermal tumor [1].

Histologically, desmoid tumors consist of elongated fibroblasts and myofibroblasts characterized by elongated, tapered cytoplasm; elongated, vesicular, typical-appearing nuclei; and multiple small nucleoli. The cells are linearly arranged and are surrounded and separated from each other by collagen [1-4,6]. These tumors show a tendency to evolve over time. Vandevenne et al [10] described three stages of evolution of desmoid tumors. In the first stage, lesions are more cellular and have fewer areas of hyalinized collagen. In the second stage, there is an increasing amount of collagen deposition in the central and peripheral areas of the tumor. In the third stage, there is an increase in the fibrous composition with a decrease in cellularity and water content [1,10].

On ultrasonography, desmoid tumors appear as well-defined lesions with variable echogenicity. The lateral borders may appear ill defined or irregular [1,7].

The CT appearance of desmoid tumors depends on their composition. They may appear homogeneous or heterogeneous and hypo-, iso-, or hyperintense compared with the attenuation of muscles. The degree of enhancement after the intravenous administration of contrast medium is variable [1,5,7,8]. In this case, the mass showed attenuation equal to that of muscle, but after the intravenous administration of contrast medium, mild enhancement was demonstrated even in the delayed images.

Magnetic resonance imaging (MRI) features of desmoid tumors also show wide variability depending on the stage they are imaged. Characteristic MRI findings include poor margination, low signal intensity on T1-weighted images and heterogeneity on T2-weighted images, and variable contrast enhancement. Low T2 signal intensity bands are characteristic and represent foci of high concentrations of collagen deposition [1,5,7].

Definitive diagnosis must be established with histopathologic analysis [1].

Wide local excision followed by reconstruction of the defect is the treatment of choice. Full-thickness resection of the tumor-containing abdominal wall with a grossly negative margin has to be performed when the lesion closely approximates or involves the peritoneum. Intraperitoneal organs or adjacent bony structures involved by tumor must be resected as well. Incomplete tumor removal or involved excision margins may lead to local recurrence [1-6].

The recurrence rate of desmoid tumors is 20% to 77% depending on the location, extent and completeness of the initial resection. Abdominal wall desmoid tumors have a significantly lower recurrence rate. Their recurrence is 20% to 30% and usually becomes evident within six months after excision or in connection with subsequent gestations or deliveries. Metastatic disease has not been reported with desmoid tumor [1,3,4,6-8].

Radiation therapy is used in patients with inoperable tumors, local recurrences or incompletely excised lesions. Chemotherapy and endocrine therapy have also been used to treat desmoid tumors in patients in whom resection is technically impossible because of a widespread tumor infiltration [1,2,4,5].

## Conclusion

The combination of features, such as the history of previous surgery, the age and sex of the patient, the location of the mass within the anterior abdominal wall and the imaging features, make desmoid tumor a strong primary diagnostic consideration even if it is a rare entity and especially in men. The treatment approach remains aggressive and includes complete surgical resection. Repair of abdominal wall defects can be sufficiently achieved with prosthetic mesh reconstruction with excellent functional results.

## Abbreviations

CT: computed tomography; FAP: familial adenomatous polyposis; MRI: magnetic resonance imaging.

## Consent

Written informed consent was obtained from the patient for publication of this case report and accompanying images. A copy of the written consent is available for review by the Editor-in-Chief of this journal.

## Competing interests

The authors declare that they have no competing interests.

## Authors' contributions

XP performed the chart review and manuscript preparation. AE and EA carried out the operation. LP was the pathologist who examined the specimen. TC participated in manuscript preparation. All authors read and approved the final manuscript.
